# CryJ-LAMP DNA Vaccines for Japanese Red Cedar Allergy Induce Robust Th1-Type Immune Responses in Murine Model

**DOI:** 10.1155/2016/4857869

**Published:** 2016-04-30

**Authors:** Yan Su, Michael Connolly, Anthony Marketon, Teri Heiland

**Affiliations:** Department of R&D, Immunomic Therapeutics, Inc. (ITI), Rockville, MD 20850, USA

## Abstract

Allergies caused by Japanese Red Cedar (JRC) pollen affect up to a third of Japanese people, necessitating development of an effective therapeutic. We utilized the lysosomal targeting property of lysosomal-associated membrane protein-1 (LAMP-1) to make DNA vaccines that encode LAMP-1 and the sequences of immunodominant allergen CryJ1 or CryJ2 from the JRC pollen. This novel strategy is designed to skew the CD4 T cell responses to the target allergens towards a nonallergenic Th1 response. CryJ1-LAMP and CryJ2-LAMP were administrated to BALB/c mice and antigen-specific Th1-type IgG2a and Th2-type IgG1 antibodies, as well as IgE antibodies, were assayed longitudinally. We also isolated different T cell populations from immunized mice and adoptively transferred them into naïve mice followed by CryJ1/CryJ2 protein boosts. We demonstrated that CryJ-LAMP immunized mice produce high levels of IFN-*γ* and anti-CryJ1 or anti-CryJ2 IgG2a antibodies and low levels of IgE antibodies, suggesting that a Th1 response was induced. In addition, we found that CD4^+^ T cells are the immunological effectors of DNA vaccination in this allergy model. Together, our results suggest the CryJ-LAMP Vaccine has a potential as an effective therapeutic for JRC induced allergy by skewing Th1/Th2 responses.

## 1. Introduction

Japanese Red Cedar (JRC) pollen driven Japanese cedar pollinosis (JCP), a type I allergic disease, affects up to a third of Japanese people [1]. T cell responses and IgE antibodies specific for the two major allergens of JRC, CryJ1 and CryJ2, have been found in most JCP patients [2–5]. Current therapies, such as oral antihistamines, antileukotrienes, and intranasal administration of corticosteroids, only partially alleviate disease symptoms and improve patients' quality of life [6–9]. However, these treatments require daily and/or long-term administration prior to and during the JRC blossom season, which is inconvenient and costly to patients. Thus, an effective and specific immunotherapy, which has long-term effects, is extremely desired.

DNA vaccination has great potential as an effective prophylactic and therapeutic solution to JCP. DNA vaccines are bacterial plasmid vectors expressing a target protein gene for* in vivo* administration and transfection. DNA vaccines have several advantages over traditional vaccines, including low cost, ease of design and manufacture, convenience of administration, and efficacy in inducing CD4^+^ and CD8^+^ T cell immunity and humoral immune responses [10]. The concept of DNA vaccines was first established in the early 1990s [11, 12]. Since then, this technique has been studied in a variety of animal models and human clinical studies for infectious diseases, cancer, allergy, and autoimmune diseases [10, 13, 14].

Endogenously produced proteins, for example, those encoded by viruses or typical DNA vaccines, are processed in the proteasomes and then undergo MHC class I presentation [15]. Lysosomal-associated membrane protein-1 (LAMP-1) is a resident protein of the lysosome. It has been shown that inclusion of the lysosomal targeting sequences of LAMP-1 in DNA plasmids directs the immunogen from a proteasomal-class I pathway towards a lysosomal-class II pathway, thus significantly enhancing the immunogenicity of target antigens in several animal models [16–18].

Thus, in this study, we integrated the advantages of the DNA vaccine technique with the MHC II pathway targeting property of LAMP-1 and designed a novel strategy for JCP therapy. We fused the CryJ1 or CryJ2 DNA sequence with the full-length LAMP-1 and tested the immunological effects of such DNA vaccines* in vivo*. Indeed, our data suggest that a robust Th1, but not Th2, response was primed as shown by high IgG2a and low IgE titers, as well as high IFN-*γ* production. Furthermore, we found that Th1 CD4^+^ T cells were induced by DNA vaccination and that adoptive transfer of such T cells induced a strong Th1-type antibody response in recipients after protein boost. Together, our data demonstrate the efficacy of our LAMP-based DNA vaccines in inducing robust nonallergenic Th1 responses, indicating this strategy has a potential for clinical application.

## 2. Materials and Methods

### 2.1. Construct and Manufacture of CryJ-LAMP DNA Vaccines

DNA vaccines encoding recombinant CryJ1-LAMP and CryJ2-LAMP proteins were generated at Nature Technology Corporation (NTC, Lincoln, NE). The CryJ1 or CryJ2 gene was codon optimized for human usage using the GeneArt/Invitrogen online gene design software. The synthetic genes were manufactured by GeneArt/Invitrogen (Life Technologies, Grand Island, NY). The synthetic gene was inserted into the N LAMP-C LAMP gene to create N LAMP-CryJ1-C LAMP or N LAMP-CryJ2-C LAMP, which was then inserted into the expression vector NTC8382-VA1. NTC8382-VA1 is a covalently closed circular double-stranded plasmid vector. The flanking regions of the insertion site are the eukaryotic promoter (CMV) and poly-A transcriptional terminator that flank the insertion site to express LAMP fusion proteins in target cells. Plasmid is transformed into NTC4862 host cell line competent cells and selected for sucrose resistance.

To verify the expression of CryJ1-LAMP and CryJ2-LAMP plasmids in mammalian cells, 293T cells were transfected with CryJ1-LAMP or CryJ2-LAMP plasmids with a Lipofectamine® 2000 Kit (Life Technologies, Grand Island, NY). Transfection reagents Opti-MEM and Lipofectamine 2000 solution only were used as a transfection control. 48 hours later, cells were washed with PBS and cell extracts were prepared using RIPA buffer. The protein concentration was determined using a Pierce BCA kit (Life Technologies, Grand Island, NY). 1 *μ*g of cell lysate was loaded onto 4–20% TGX gels (BioRad, Hercules, California) and the gels were blotted to nitrocellulose and then blocked with KPL Detector Block (KPL, Gaithersburg, MD). Membranes were then probed with polyclonal rabbit anti-CryJ1, anti-CryJ2, or anti-human-LAMP antibody, respectively. Goat anti-rabbit-HRP (Jackson ImmunoResearch Laboratories, West Grove, PA) was used as a secondary antibody and membranes were developed with KPL LumiGlo Reserve. Images were generated by using the ChemiDoc XRS system (BioRad).

### 2.2. Construct, Expression, and Purification of CryJ1 and CryJ2 Proteins

To make the recombinant CryJ1 and CryJ2 proteins, the pVEXK-CryJ1 and CryJ2 expression vectors were constructed. The CryJ1 or CryJ2 gene was amplified by PCR and then digested with* Nde*I and* Sal*I. Then the CryJ1 or CryJ2 gene was cloned into the IPTG inducible tac promoter* E. coli* expression vector pVEXK HN-MCS-K6EE [19]. The resultant vector expresses recombinant proteins with an N-terminal 6x HN histidine tag (metal binding alterative to the his-tag for immobilized metal chelate chromatography (IMAC) affinity purification) and a protein stability and solubility enhancing C-terminus six-lysine, two-glutamic-acid-residue tag (K6EE). Recombinant clones were confirmed by restriction digest and DNA sequencing. pVEXK HN CryJ1 K6EE and pVEXK HN CryJ2 K6EE vector transfected cells were cultured in IPTG-induced LB shake flask for protein purification. Cultures were grown at 30–37°C in LB media containing 0.2% glucose and induced using 1 mM IPTG at 0.5–1.0 OD600 for 4–8 hr. Cells were then harvested, resuspended, and lysed by freeze-thawing and sonication and centrifuged. HN-tagged protein in the clarified lysate was purified on a Talon resin (Clontech Laboratories, Mountain View, CA) IMAC column. Endotoxin was quantified using the Endosafe PTS system (Charles River Laboratories, Wilmington, MA). Recombinant protein was quantified by A280 using the theoretical extinction coefficient.

### 2.3. Crude JRC Extract Preparation

Defatted JRC pollen (Greer, Lenoir, NC) was incubated with 20 volumes (wt/vol) of NH_4_CO_3_ solution (0.125 M) overnight at 4°C. After centrifugation at 2500 g for 20 minutes at 4°C, the supernatant was collected and the concentration of JRC extract was determined by using a Pierce BCA kit.

### 2.4. Animals and DNA Vaccination Regimens

BALB/c mice (Harlan, Frederick, MD) were ordered and hosted in our animal facility and entered experiments when they became 5-6 weeks old. We immunize mice three times weekly with 50 *μ*g DNA plasmid through IM or ID injection or ID using a CO_2_ pressured Biojector (Bioject Medical Technologies, Tigard, OR). Four weeks after the last DNA immunization, mice were boosted with 5 *μ*g recombinant protein or 100 *μ*g JRC extract in the presence of alum adjuvant by i.p. injection. Because one exposure of allergens elicited a trace amount of IgE antibody, mice were boosted again with allergens in the presence or absence of alum adjuvant as indicated. Mice were bled at the indicated times and plasma or serum samples were isolated for antibody detection. As donors of splenocytes, mice were immunized with three doses of CryJ1-LAMP and/or CryJ2-LAMP plasmids and then sacrificed two weeks after the last dose (10-11 weeks old). Mice were immunized, bled, and sacrificed at the end of study as indicated in each individual experiment. In this study, brief and transient pain/discomfort for the mice after each treatment was as expected. Mice were sedated with carbon dioxide (70%) and oxygen (30%) for approximately 30 seconds to alleviate pain prior to the Bioject injection. All mice were sacrificed by carbon dioxide anesthesia and quick cervical dislocation to minimize animal suffering.

### 2.5. T Cell Purification and Adoptive Transfer

Anti-mouse CD4, CD8, and B220 antibodies were purchased from BioLegend (San Diego, CA). Spleen T cells were purified by depleting B220^+^, CD4^+^, or CD8^+^ cells to get the whole T, CD8^+^ T, or CD4^+^ T cell populations. Freshly isolated splenocytes were incubated with magnetic beads which were precoated with anti-mouse B220 alone, B220 plus anti-CD8, or B220 plus anti-CD4 mAbs for one hour. Then target cell populations were isolated by negative selection through a magnetic separator. The purity was tested by flow cytometry and more than 85% purity was achieved. Isolated T cells were adoptively transferred into naïve BALB/c mice via tail vein injection. 1 × 10^7^ whole T or CD4^+^ T cells and 7 × 10^6^ CD8^+^ T cells were transferred per mouse on the same day of purification.

### 2.6. IgG1, IgG2a, and IgE Antibody Detection by ELISA

ELISA plates (MaxiSorp for IgG and Immulon 4HBX for IgE) were coated with 5 *μ*g/mL recombinant protein or 40 *μ*g/mL crude JRC extract overnight and then blocked with 2% BSA in PBS. Serum samples were diluted 1 : 100- or 1 : 1000-fold in 1% BSA in PBS and then a 1 : 3 serial dilution was made. To detect IgE, sera were treated with Agarose-Protein G (Thermo Fisher Scientific, Rockford, IL) for 50 minutes and then 1 : 20 diluted samples were loaded to ELISA plates. Samples were detected with goat anti-mouse IgG1-HRP, goat anti-mouse IgG2a-HRP (Southern Biotech, Birmingham, AL), or rat anti-mouse-IgE-biotin (R35-118, BD Pharmingen, San Jose, CA) followed by Pierce Streptavidin-HRP (Thermo Fisher Scientific, Rockford, IL). Reaction was developed with SureBlue TMB Substrate (KPL, Gaithersburg, MD) and stopped with TMB Stop Solution (KPL, Gaithersburg, MD). Plates were read (OD_450_) by using Epoch ELISA reader (BioTek, Winooski, VT). Average background (PBS only) was calculated, and samples which have OD_450_ value more than 2 *∗* average background are considered as positive. The dilutions of such samples are determined as the endpoint titers.

### 2.7. Regulatory T Cell Depletion and IL-10 Blocking* In Vivo*

Anti-mouse CD25 (PC61.5) and rat IgG1 isotype control antibodies were purchased from eBiosciences (San Diego, CA). Mice were i.p. injected with 100 *μ*L of anti-CD25 or isotype control antibody 4 days before the first DNA vaccination. The depletion of CD4^+^CD25^+^ regulatory T cells was confirmed three days later by checking the CD4^+^CD25^+^ Tregs in peripheral blood. After red blood cell lysis, cells were stained with CD4-FITC and CD25-APC and then analyzed by flow cytometry (BD Accuri C6). The IL-10 signaling was blocked as previously described [20, 21]. Briefly, mice were i.p. injected with 0.5 mg of rat anti-mouse IL-10 receptor mAb (clone 1B1.3a, BioLegend, San Diego, CA) on days 0, 7, 14, 21, 28, and 34.

### 2.8. Cytokine Detection by ELISPOT

IL-4 and IFN-*γ* produced by splenocytes were examined by using ELISPOT at the end of study. Spleens were processed to make single cell solution after red blood cell lysis. 5 × 10^5^ cells per well were added to anti-mouse IL-4 or IFN-*γ* antibody (Invitrogen, Carlsbad, CA) coated PVDF membrane ELISPOT plates (EMD Millipore, Billerica, MA). Cells were cultured with or without 1 *μ*g/mL CryJ2 protein. Cells incubated with ConA were used as a positive control. 48 hours later, plates were washed and then incubated with biotin-conjugated IL-4 or IFN-*γ* detection antibody followed by Streptavidin-AP (Southern Biotech, Birmingham, AL). Plates were developed with BCIP substrate (KPL) and spots were counted with Advanced Imaging Devices GmbH system (Strassberg, Germany).

### 2.9. Basophil Test

Basophil test and IL-4 production detection followed a method previously described by Torrero et al. with minor modification [22]. CD200R was used as a marker of basophil activation and IL-4 was used to evaluate basophil function. 250 *μ*L heparinized whole blood was collected from each mouse. Two samples within group were pooled and then 1 : 1 diluted with DMEM medium. 500 uL diluted blood samples were incubated with or without 1 *μ*g/mL CryJ2 protein at 37°C. One hour later, cells were treated with protein transport inhibitor Golgi-Plug (BD Biosciences, San Jose, CA) and then continued to be cultured for 100 minutes. Cells were stained with IgE-FITC, CD4-PerCP, B220-PerCP, and CD200R-PE (BioLegend, San Diego, CA) for 30 minutes and treated with RBC lysis/fixation buffer (BioLegend). Cells were then intracellularly stained with anti-mouse IL-4-APC (BioLegend) for 30 minutes and analyzed by flow cytometry (BD Accuri C6). Basophils were defined as IgE^+^CD4^−^B220^−^ cells. For basophil activation assays, cut-off gates for CD200R-PE and IL-4-APC positivity were established using the fluorescence-minus-one (FMO) technique.

### 2.10. Statistical Analysis

Data are represented as mean ± SEM. Statistical analyses were performed by using Prism 6 software (GraphPad Software, La Jolla, CA). Data were analyzed by one-way ANOVA followed by Tukey's test for multiple comparisons. *p* values below 0.05 were considered to indicate a statistically significant difference.

## 3. Results

### 3.1. Evaluation of the* In Vivo* Effects of CryJ-LAMP Vaccines by Different Delivery Routes

**Figure 1:**
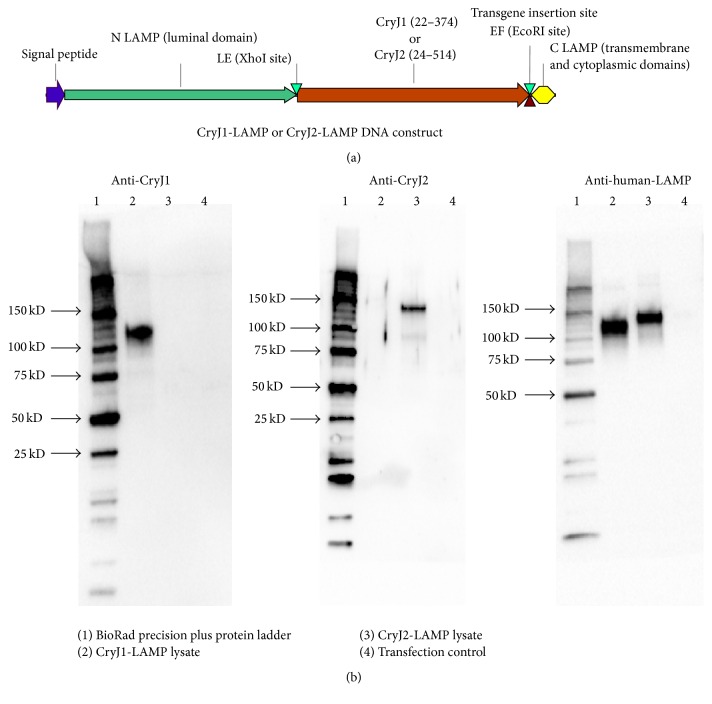
CryJ-LAMP DNA construct and expression* in vitro*. (a) Plasmid construction of LAMP Vaccine. CryJ1-LAMP and CryJ2-LAMP plasmids were generated by inserting synthetic CryJ1 or CryJ2 gene into the N LAMP-C LAMP gene to create N LAMP-CryJ1-C LAMP or N LAMP-CryJ2-C LAMP. (b) 293T cells were transfected with CryJ1-LAMP or CryJ2-LAMP. Transfection reagents Opti-MEM and Lipofectamine 2000 solution only were used as a transfection control. Cell lysates were examined for expression of CryJ1-LAMP or CryJ2-LAMP by using antibodies against CryJ1 (left), CryJ2 (middle), or human-LAMP (right), respectively.

**Figure 2:**
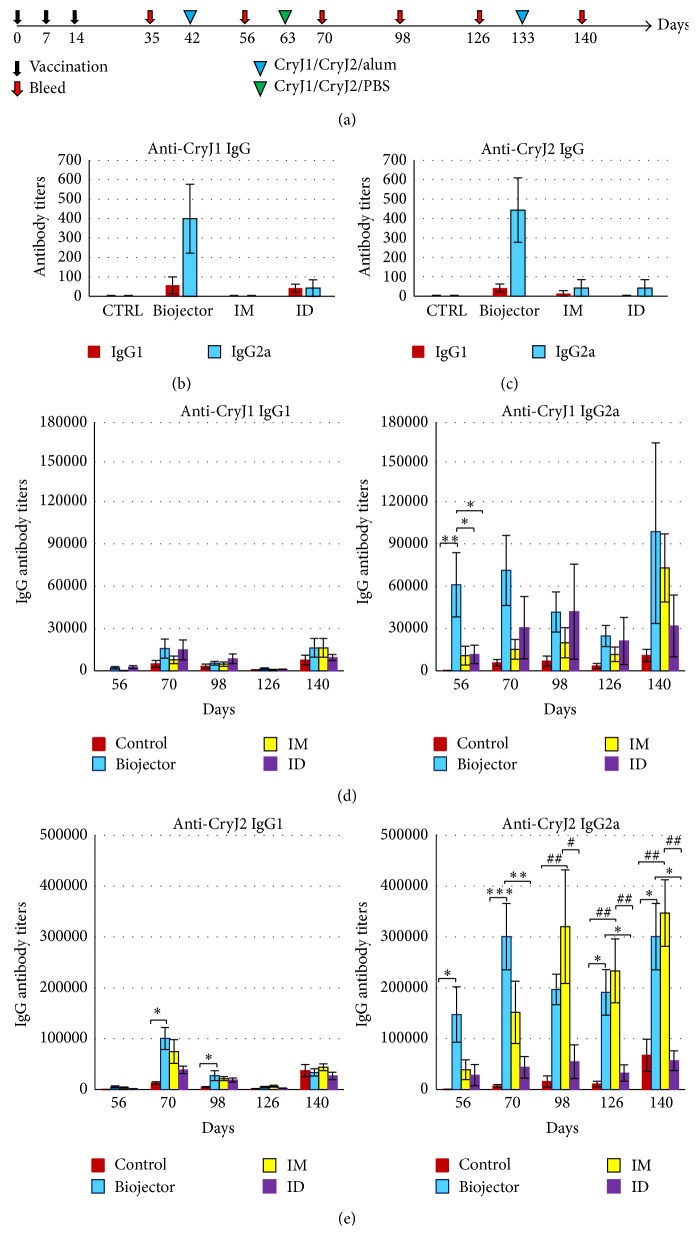
CryJ1-LAMP and CryJ2-LAMP Vaccines induce a robust immune response* in vivo*. (a) Schematic view of vaccination protocol. Four groups of mice (*n* = 7) were immunized with a mixture of 50 *μ*g CryJ1-LAMP and 50 *μ*g CryJ2-LAMP plasmids by intramuscular (IM), intradermal (ID), or needle-free CO_2_ pressure Biojector injection. Control vector was delivered by ID injection. Mice were boosted with a mixture of 5 *μ*g recombinant CryJ1 and 5 *μ*g CryJ2 in the presence of alum adjuvant as indicated. Mice were bled as indicated. Anti-CryJ1 (b) and anti-CryJ2 (c) IgG1 and IgG2a antibodies after three DNA injections were tested on day 35 by endpoint ELISA method. Anti-CryJ1 (d) and anti-CryJ2 (e) IgG1 and IgG2a antibodies after protein boosts were assessed and summarized. This is a representative of two similar studies. All data are shown as mean ± SEM. Comparisons between Biojector and control, IM, or ID, ^*∗*^ *p* < 0.05, ^*∗∗*^ *p* < 0.01, and ^*∗∗∗*^ *p* < 0.001; comparisons between IM and control or ID, ^#^ *p* < 0.05 and ^##^ *p* < 0.01.

We generated two DNA plasmids, CryJ1-LAMP and CryJ2-LAMP (Figure 1(a)). We first verified the expression of DNA vaccines in mammalian 293T cells. Expression of such constructs in 293T cell lysates was shown in Figure 1(b). Then, we studied the* in vivo* immunological effects of the CryJ1-LAMP and CryJ2-LAMP DNA vaccines. For this, we compared three administration routes, intradermal (ID) and intramuscular (IM) injections and a needle-free Biojector delivery. Four groups of mice were immunized with a control vector DNA (ID) or 1 : 1 mixed CryJ1-LAMP and CryJ2-LAMP DNA vaccines through ID, IM, or Biojector, respectively. A schematic overview of the experimental procedure is shown in Figure 2(a). We examined production of Th2-associated IgG1 and Th1-associated IgG2a antibodies against CryJ1 or CryJ2 protein. As shown in Figures 2(b)-2(c), three doses of DNA vaccines induced minimal levels of IgG1 or IgG2a antibodies against CryJ1 and/or CryJ2 protein by IM or ID injection. In contrast, Biojector delivered DNA vaccines elicited a moderate level of IgG2a anti-CryJ1 and anti-CryJ2 antibodies. At this time, no anti-CryJ1 or anti-CryJ2 IgE antibodies were induced in any group (data not shown).

To test the immunological effects of CryJ-LAMP Vaccines after allergen exposure, vaccinated mice were boosted with recombinant CryJ1 and CryJ2 proteins through intraperitoneal injection (i.p.) with alum adjuvant. The production of serum anti-CryJ1 and anti-CryJ2 antibodies was assayed longitudinally. As shown in Figures 2(d) and 2(e), all mice that received DNA vaccinations exhibited more IgG, especially Th1-associated IgG2a, antibody production than the controls. It is worth noting that the ratios of mean IgG2a-to-IgG1 antibody titers in CryJ-LAMP vaccinated mice (particularly by Biojector and IM injection) are higher than those of control mice (S1. Figure in Supplementary Material available online at http://dx.doi.org/10.1155/2016/4857869). These results suggest a dominant Th1-type immune response was induced by CryJ-LAMP Vaccines. In terms of the Th2-associated IgG1 subclass, there is no difference among the three delivery routes. CryJ-LAMP Vaccines that were delivered by Biojector rapidly primed high titers of both anti-CryJ1 and anti-CryJ2 IgG2a antibodies after CryJ1/CryJ2 protein boost (as shown on days 56 and 70). Furthermore, production of both anti-CryJ1 and anti-CryJ2 IgG2a antibodies in the Biojector group remained high and consistent until the end of this study. IM treated mice exhibited significant higher IgG2a titers to CryJ2, but not to CryJ1 protein, 12 weeks after the last DNA vaccine dose (day 98 and after). These indicate that the IM injection needs a longer time to prime the Th1 response. ID delivered CryJ-LAMP Vaccines only elicited strong IgG2a production to CryJ1, but not to CryJ2. Anti-CryJ1 and anti-CryJ2 IgE antibodies were also tested. Biojector or IM vaccinated mice exhibited lower production of anti-CryJ1 or anti-CryJ2 IgE antibody than the controls, whereas the ID vaccinated mice showed no difference from the controls (data not shown).

**Figure 3:**
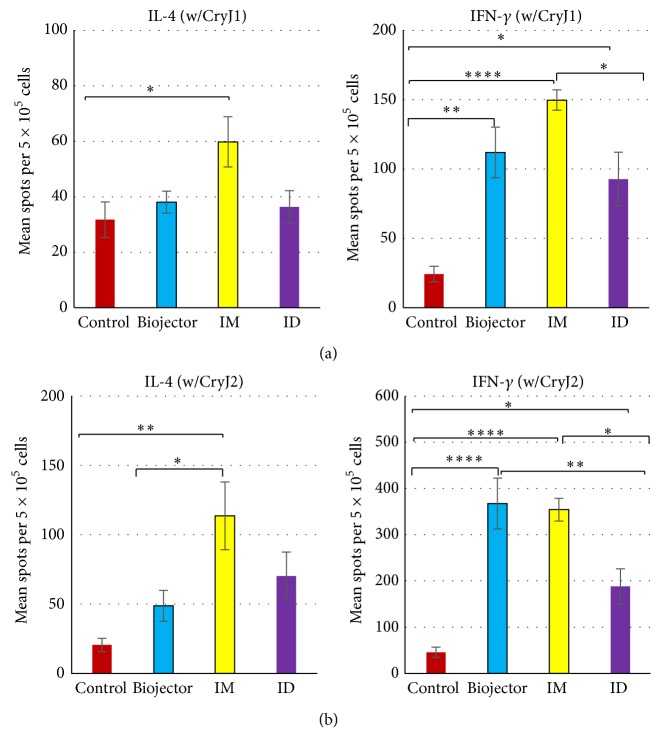
CryJ1-LAMP and CryJ2-LAMP Vaccines induce a strong Th1 cellular response. Vaccination protocol is described in Figure 2. At the end of study, freshly isolated splenocytes were treated with 1 *μ*g/mL CryJ1 (a) or CryJ2 (b) protein for 48 hours. IL-4 and IFN-*γ* production were detected by ELISPOT. All data are shown as mean ± SEM. ^*∗*^ *p* < 0.05, ^*∗∗*^ *p* < 0.01, and ^*∗∗∗∗*^ *p* < 0.0001.

At the end of this study, splenocytes were cultured with CryJ1 or CryJ2 protein and the production of Th1-associated cytokine IFN-*γ* and Th2-associated cytokine IL-4 was examined by ELISPOT (Figure 3). CryJ-LAMP Vaccines delivered through all three routes induced significantly higher levels of CryJ1- and CryJ2-specific IFN-*γ* spot-forming cells than the controls (Figures 3(a) and 3(b), right). Biojector and IM injection treated mice exhibited similar levels of CryJ2- and/or CryJ1-specific IFN-*γ* spot-forming cells, which are higher than that of the ID injection. Biojector and ID treated mice have similar levels of CryJ1-specific IL-4 spot-forming cells to the control mice (Figure 3(a), left). Both Biojector and ID groups showed more CryJ2-specific IL-4 spot-forming cells than the control mice (Figure 3(b), left; no significant difference). However, IM treated mice showed significantly higher levels of both CryJ1- and CryJ2-specific IL-4 spot-forming cells than the control mice (Figures 3(a) and 3(b), left), suggesting an induction of a Th1 predominant but Th1/Th2 mixed response in this group. Together, these results confirmed that CryJ-LAMP Vaccines induced a predominant Th1 cellular response, particularly through Biojector delivery.

These data indicated that ID injection is the least efficient method of vaccination under the tested protocol. As IM or ID injection is commonly used in DNA vaccine studies, we further compared Biojector and ID injection in a modified vaccination protocol with four doses of vaccination. Vaccination by two methods elicited similar levels of JRC specific IgG2a antibody production if mice were boosted with JRC extract 14 weeks after the first DNA vaccination (S2. Figure). These suggest that ID injection via needle is effective but requires a longer time to elicit a strong antibody response. Taken together, our results indicate that the Biojector delivery rapidly primes the animals, particularly in inducing the IgG2a antibody production. Therefore, we utilized the Biojector delivery in our subsequent studies to shorten the experiment. Furthermore, our data demonstrated that the CryJ-LAMP DNA vaccines induced high levels of IgG2a but moderate levels of IgG1 antibodies, as well as high levels of IFN-*γ* production, suggesting an induction of Th1 responses or a Th1 skewing.

### 3.2. CryJ-LAMP Vaccines Affect IL-4 Production by Basophils

**Figure 4:**
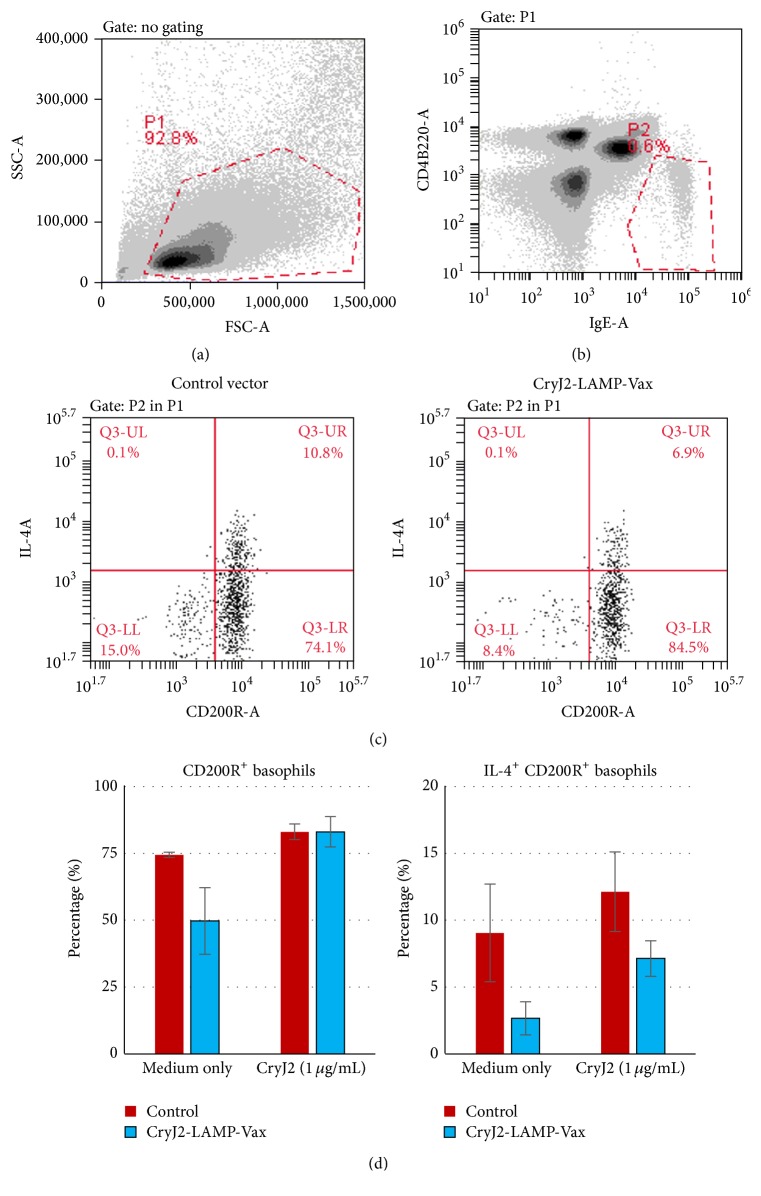
CryJ2-LAMP vaccination affects IL-4 production by basophils. Two groups of mice (*n* = 6) were immunized with 50 *μ*g CryJ2-LAMP plasmid or a control vector by Biojector injection on days 0, 7, and 14. Mice were boosted with 5 *μ*g CryJ2 on day 42. On day 77, peripheral blood was assayed for basophils activation and IL-4 production. (a) Cells are gated by size. (b) IgE^+^CD4^−^B220^−^ cells are defined as basophils. (c) Expression of IL-4 and CD200R in IgE^+^CD4^−^B220^−^ cells from two representative samples from control (*left*) or CryJ2-LAMP (*right*) group is shown. (d) Analysis of percentages of CD200R^+^/IgE^+^CD4^−^B220^−^ cells (*left*) and IL-4^+^/CD200R^+^IgE^+^CD4^−^B220^−^ cells (*right*) is shown. All data are shown as mean ± SEM.

Basophils are major effector cells of allergic responses. To study the effects of CryJ-LAMP DNA vaccines on basophils, two groups of mice were immunized with a control vector or CryJ2-LAMP Vaccine three times on days 0, 7, and 14. Then mice were boosted with CryJ2 protein on day 42. On day 77, mice were bled for basophil test. Peripheral blood samples were stimulated with CryJ2 protein* in vitro* for 3 hours and IL-4 production was analyzed by intracellular staining. As shown in Figures 4(a) and 4(b), IgE^+^CD4^−^B220^−^ cells were gated as basophils. CD200R and IL-4 were used as markers of basophil activation and function, respectively (Figure 4(c)). In the absence of CryJ2* in vitro* stimulation (medium only), CryJ2-LAMP group exhibited fewer CD200R^+^ basophils than the control. When stimulated with CryJ2 protein, both groups showed similar percentage of CD200R^+^ cells in IgE^+^CD4^−^B220^−^ population (Figure 4(d), left). However, the CryJ2-LAMP group showed lower percentages of IL-4-producing CD200R^+^ basophils with or without* in vitro* CryJ2 stimulation (Figure 4(d), right). These results suggest that CryJ2-LAMP vaccination might suppress basophil activation and/or function.

### 3.3. Induction of Antigen-Specific T Cell Memory* In Vivo* by CryJ-LAMP Vaccines

We propose that, following vaccination with CryJ1-LAMP and CryJ2-LAMP DNA vaccines, antigen-presenting cells, such as dendritic cells, express the fusion protein and then present the processed epitopes to CD4^+^ T cells, which help B cells produce protective IgG2a antibodies. To test the role of T cells in the mechanisms of action (MOA) of the CryJ-LAMP Vaccines, we performed a series of T cell adoptive transfer studies. We first transferred the entire T cell population, isolated from CryJ1-LAMP or CryJ2-LAMP vaccinated mice, into naïve recipients. Animals were boosted with a mixture of three recombinant proteins, including CryJ1, CryJ2, and a flea antigen (rFlea) as an irrelevant control. We examined serum anti-CryJ1, anti-CryJ2, or anti-rFlea IgG antibody, respectively. As shown in S3. Figure, mice that received CryJ1- or CryJ2-specific T cells exhibited higher titers of anti-CryJ1 or anti-CryJ2 antibodies, respectively. The titers of IgG2a are 3–6-fold higher than those of IgG1. There was no difference in the production of anti-rFlea antibodies in all groups. Therefore, these results confirmed that DNA vaccination induced antigen-specific T cell memory.

**Figure 5:**
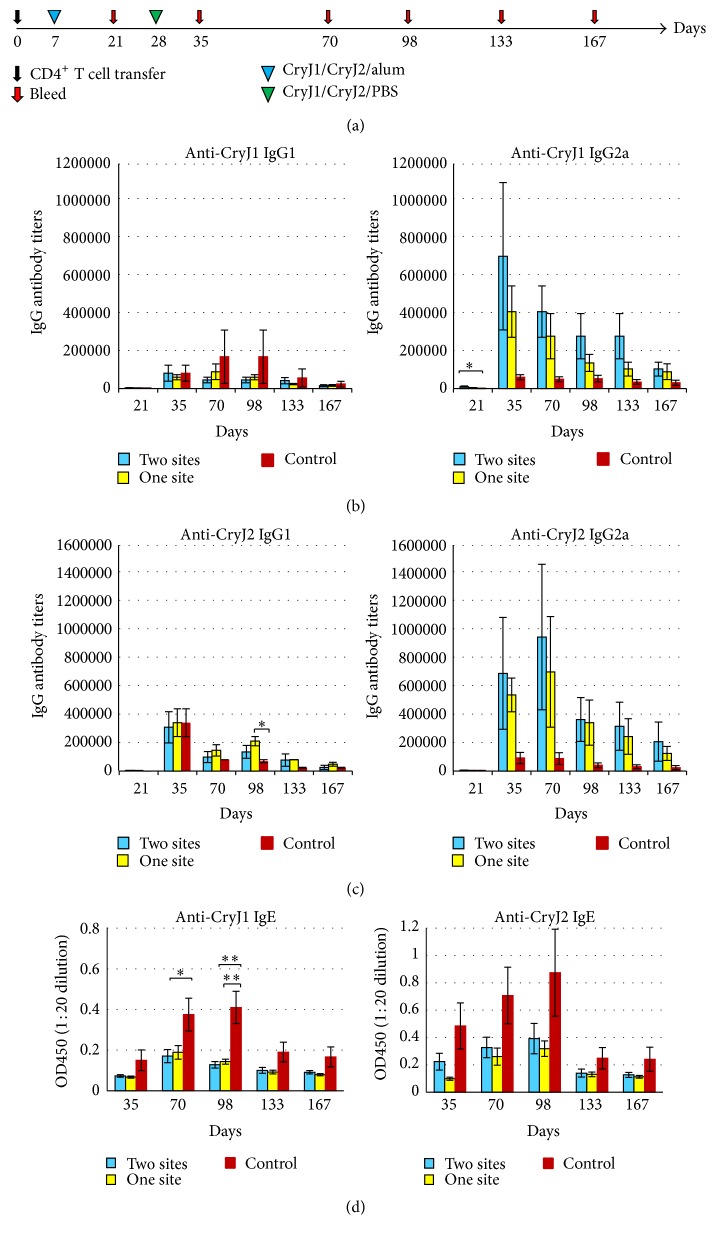
DNA vaccination induces CD4^+^ T cell memory response. (a) Schematic view of vaccination protocol. Three groups of mice were immunized with control vector, 50 *μ*g CryJ1-LAMP and 50 *μ*g CryJ2-LAMP in one injection (one site), or the same amount of CryJ1-LAMP and CryJ2-LAMP separately on flanks (two sites) three times (weekly) by Biojector injection. Two weeks after the last injection, mice were sacrificed and spleens were removed. CD4^+^ T cells were isolated and 1 × 10^7^ CD4^+^ T cells per mouse were transferred into naïve mice accordingly (*n* = 5) on day 0 by intravenous injection. Recipients were then boosted with 5 *μ*g CryJ1 and 5 *μ*g CryJ2 and bled as indicated. Anti-CryJ1 (b) and anti-CryJ2 (c) IgG1 and IgG2a antibodies were analyzed by ELISA. (d) Anti-CryJ1 and anti-CryJ2 IgE antibodies were determined by ELISA and depicted are results of OD_450_ values at a serum dilution of 1 : 20. The OD_450_ values of prebleed samples are below 0.06. All data are shown as mean ± SEM. ^*∗*^ *p* < 0.05 and ^*∗∗*^ *p* < 0.01.

Then, we asked whether T cell memory is induced in CD4^+^ T cells by transferring CD4^+^ T cells from CryJ-LAMP vaccinated mice into naïve recipients. In addition, for the purpose of optimization for clinical application, we compared two vaccination methods, administrating two CryJ-LAMP DNA vaccines together or separately. CryJ1- and CryJ2-specific T cells were generated by immunizing mice three times, once weekly, with CryJ1-LAMP and CryJ2-LAMP. One group was given mixed CryJ1-LAMP and CryJ2-LAMP in a single site (so named as one site) and another group was given the two vaccines in separated sites (two sites). Two weeks after the last DNA vaccination, splenic CD4^+^ T cells were purified and then transferred into naïve recipients. CD4^+^ T cells from mice immunized with a control vector were used as controls. Recipient mice were boosted and bled as indicated in Figure 5(a). Anti-CryJ1 and anti-CryJ2 IgG and IgE antibodies were measured. As shown in Figures 5(b) and 5(c), both groups (one and two sites) exhibited higher anti-CryJ1 or anti-CryJ2 IgG2a antibody production than that of the controls. Anti-CryJ1 or anti-CryJ2 IgG1 antibody was also produced, but at a level lower than that of IgG2a. Although no significant difference was reached when comparing with the IgG2a production, the profiles of IgG2a-to-IgG1 ratios clearly showed a difference between two CryJ-LAMP vaccinated groups and the control group (S4. Figure). The high levels of IgG2a antibody production lasted up to 24 weeks, when we terminated this study. Moreover, both groups exhibited lower anti-CryJ1 and/or anti-CryJ2 IgE responses than those of the controls (Figure 5(d)). There was no significant difference between one-site and two-site groups in both IgG and IgE production, suggesting delivery of the bivalent vaccines in one injection is as effective as separate vaccination. Clearly, these results suggest that CD4^+^ T cell memory is induced by DNA vaccination.

### 3.4. Immunological Effects of CryJ-LAMP Vaccines through CD4^+^, but Not CD8^+^, T Cells

**Figure 6:**
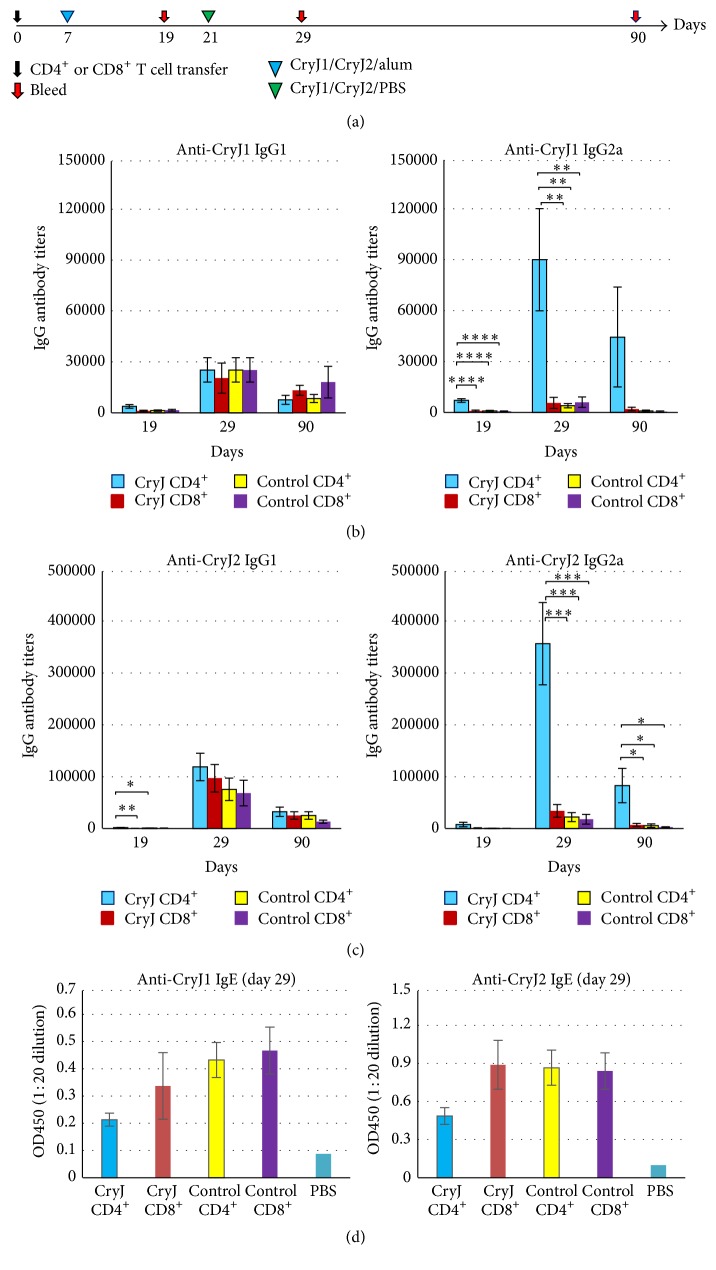
Transfer of CD4^+^, but not CD8^+^, T cells primes a Th1 antibody response in recipients. (a) Schematic view of vaccination protocol. Two groups of mice were immunized with control vector or mixed 50 *μ*g CryJ1-LAMP and 50 *μ*g CryJ2-LAMP three times (weekly). Two weeks after the last injection, mice were sacrificed and spleens were removed. CD4^+^ or CD8^+^ T cells were isolated and 1 × 10^7^ CD4^+^ or 7 × 10^6^ CD8^+^ T cells per mouse were transferred into naïve mice accordingly (*n* = 5) on day 0 through tail vein. Recipients were subsequently boosted with 5 *μ*g CryJ1 and 5 *μ*g CryJ2 and bled as indicated. Anti-CryJ1 (b) and anti-CryJ2 (c) IgG1 and IgG2a antibodies were analyzed by ELISA. (d) IgE antibodies were determined by ELISA and depicted are results of OD_450_ values at a serum dilution of 1 : 20. All data are shown as mean ± SEM. ^*∗*^ *p* < 0.05, ^*∗∗*^ *p* < 0.01, ^*∗∗∗*^ *p* < 0.001, and ^*∗∗∗∗*^ *p* < 0.0001.

One advantage of a DNA vaccine is its efficacy of inducing strong CD8^+^ T cell response. In the presence of LAMP-1 protein, DNA vaccine encoded, endogenously produced antigens are theoretically directed to the lysosomes/endosomes. However, we could not preclude the possibility of proteasome processing and the subsequent MHC class I presentation in the antigen-presenting cells. Indeed, it has been previously found that a DNA vaccine encoding the LAMP-1 and HIV* gag* protein induced both strong CD4^+^ and CD8^+^ T cell responses in mice [23, 24]. The LAMP/*gag* plasmid activated Gag-specific CD8^+^ T cells, characterized by high IFN-*γ* production and enhanced Gag-specific cell killing. Gag-specific antibodies were induced in immunized mice as well. Thus, we next examined whether CD8^+^ T cells are involved in the MOA of CryJ-LAMP Vaccines. We isolated either CD4^+^ or CD8^+^ T cells from CryJ1-LAMP and CryJ2-LAMP vaccinated mice and then transferred them into naïve mice followed by a CryJ1/CryJ2 protein boost (Figure 6(a)). Mice that received CryJ1/CryJ2 CD4^+^ T cells produced significantly higher titers of anti-CryJ1 and anti-CryJ2 IgG2a antibodies than the other three groups, including recipients of CryJ1/CryJ2 CD8^+^ T cells (Figures 6(b) and 6(c)). The IgG1 titers against either CryJ1 or CryJ2 are comparable among groups. In addition, the profile of IgG2a-to-IgG1 ratios from the CryJ1/CryJ2 CD4^+^ T cells recipients is distinct from the other three groups (S5. Figure). Moreover, recipients of CryJ1/CryJ2 CD4^+^ T cells exhibited lower CryJ1- or CryJ2-specific IgE production than the other three groups (Figure 6(d)). Mice that received CryJ1/CryJ2 CD8^+^ T cells did not show any difference from the controls.

### 3.5. Role of IL-10 and Tregs in the MOA of the CryJ-LAMP Vaccines

**Figure 7:**
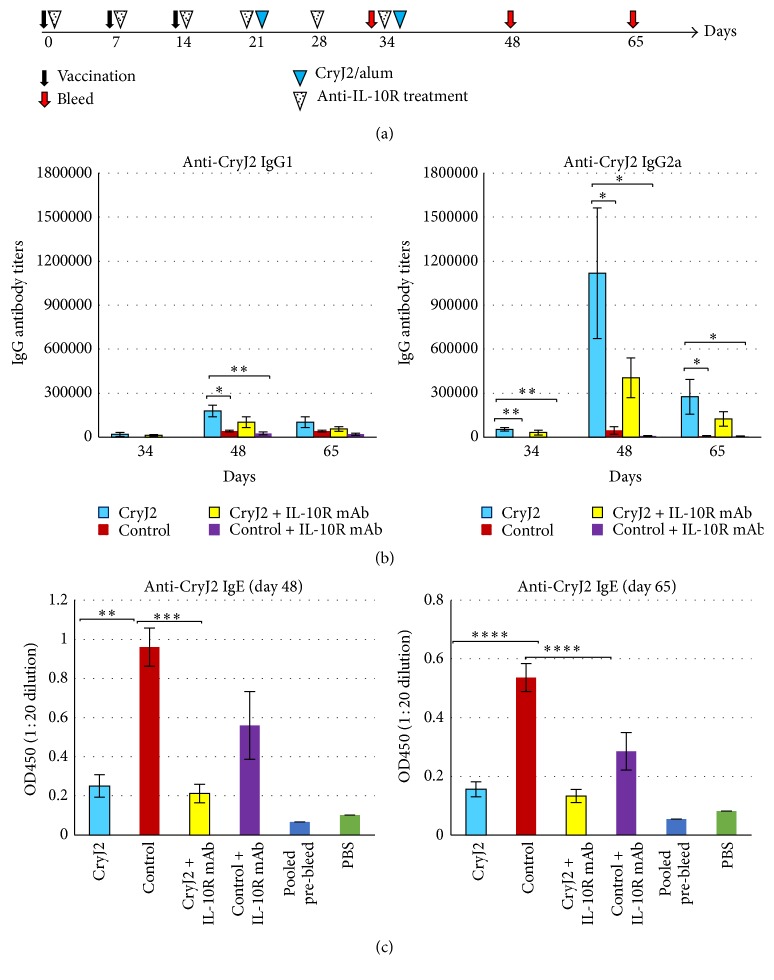
IL-10R blockage does not change the priming of Th1 responses upon DNA vaccination. (a) Schematic view of vaccination protocol. Four groups of mice (*n* = 5) were immunized with 50 *μ*g control vector or 50 *μ*g CryJ2-LAMP. Two groups were treated weekly with 0.5 mg anti-IL-10R mAb (clone 1B1.3a) six times by i.p. injection. Then recipients were boosted with 5 *μ*g CryJ2 and bled as indicated. (b) Anti-CryJ2 IgG1 and IgG2a antibodies were analyzed by ELISA. (c) Anti-CryJ2 IgE antibodies were determined by ELISA and depicted are results of OD_450_ values at a serum dilution of 1 : 20. All data are shown as mean ± SEM. ^*∗*^ *p* < 0.05, ^*∗∗*^ *p* < 0.01, ^*∗∗∗*^ *p* < 0.001, and ^*∗∗∗∗*^ *p* < 0.0001.

Regulatory T cells (Tregs) play an essential role in immune regulation and their activity is related to IL-10, a key cytokine in controlling immune responses. It is possible that CryJ-LAMP Vaccines induce activation of Tregs. Thus, we first investigated the potential role of IL-10/Tregs in the MOA by blocking IL-10 signaling. Mice were treated with anti-IL-10 receptor monoclonal antibody (anti-IL-10R mAb) weekly during and after CryJ2-LAMP DNA vaccination (Figure 7(a)). As shown in Figure 7(b), anti-IL-10R mAb treated mice produced both IgG1 and IgG2a anti-CryJ2 antibodies similarly to those of untreated mice. The patterns of IgG2a-to-IgG1 ratios from CryJ2-LAMP vaccinated mice (with and without IL-10R blockage) are similar; both differ from the control groups (S6. Figure). These results suggest that anti-IL-10R mAb treatment does not reverse the Th1 skewing. We also tested IgE production, and CryJ2-LAMP immunized mice with or without anti-IL-10R mAb treatment exhibited significantly lower IgE production (Figure 7(c)). It is worth noting that IL-10R blocked mice produce less IgE, IgG1, or IgG2a antibodies than untreated mice. These results suggest that anti-IL-10R blockage might have a systematic effect on the immune system; however, it does not change the Th1/Th2 skewing mediated by DNA vaccination.

**Figure 8:**
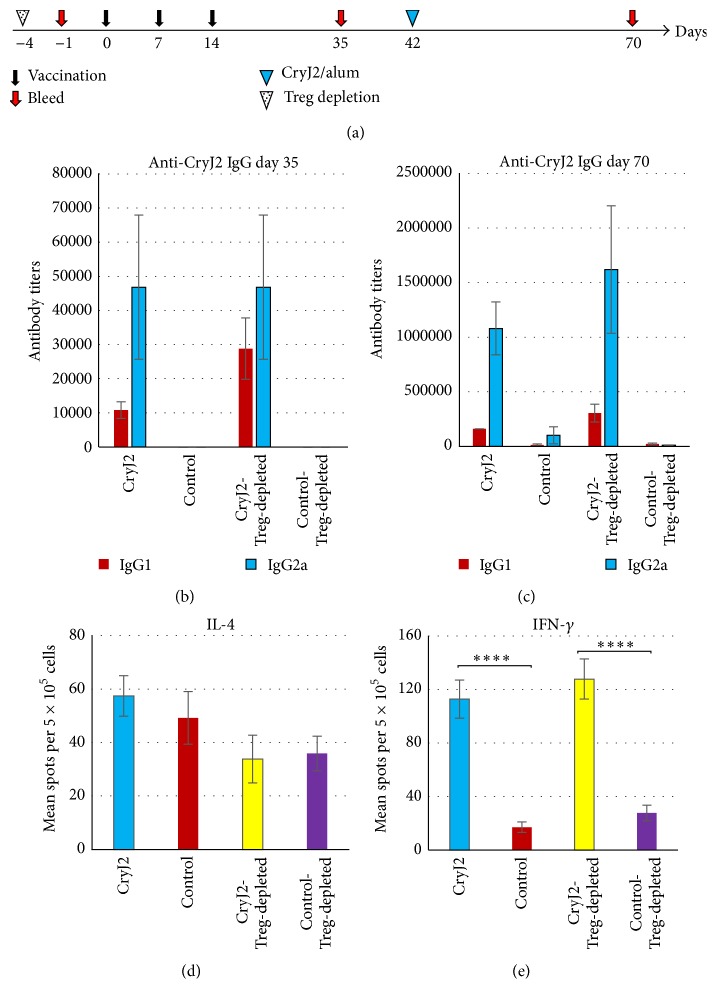
Treg depletion does not change the priming of Th1 responses upon DNA vaccination. (a) Schematic view of vaccination protocol. Tregs were depleted prior to DNA vaccination. Treg-depleted and control antibody treated mice (*n* = 6) were immunized with 50 *μ*g control vector or 50 *μ*g CryJ2-LAMP, respectively. After three DNA vaccinations, recipients were boosted with 5 *μ*g CryJ2 and bled as indicated. CryJ2-specific IgG1 and IgG2a antibodies on day 35 (b) and day 70 (c) were analyzed by ELISA. At the end of this study, IL-4 (d) and IFN-*γ* (e) production from splenocytes* in vitro* culture (1 *μ*g/mL CryJ2) were analyzed by ELISPOT. This is a representative of three similar studies. All data are shown as mean ± SEM. ^*∗∗∗∗*^ *p* < 0.0001.

To further investigate the role of Tregs in the MOA, we treated mice with anti-CD25 mAb to deplete natural CD4^+^CD25^+^ Tregs before CryJ2-LAMP DNA vaccination. Treg depletion in peripheral blood samples was validated before vaccination (data not shown). As shown in Figure 8, Treg depletion has no effects on CryJ2-specific IgG2a antibody production. Before CryJ2 protein boost (day 35), the IgG1 level in Treg-depleted mice is higher than that of CryJ2-LAMP immunized, untreated mice, possibly due to the lack of suppression from Tregs (Figure 8(b)). However, after protein boost, both Treg-depleted and control mice exhibited similar profiles of IgG production (Figure 8(c)). Splenocytes were cultured with CryJ2 protein* in vitro* for testing IL-4 and IFN-*γ* production. All four groups exhibited similar levels of IL-4 spot-forming cells (Figure 8(d)). CryJ2-LAMP vaccinated mice (Treg-depleted or not) showed similar levels of IFN-*γ* spot-forming cells, indicating that depletion of Tregs has no effects on induction of Th1 response (Figure 8(e)). Taken together, these results suggest that the immunological effects of CryJ-LAMP Vaccines or the Th1/Th2 skewing are independent of natural Tregs and/or IL-10 pathway.

## 4. Discussion 

Recently, ITI's DNA vaccine CryJ2-LAMP-Vax met its safety and immunological endpoints in a Phase IA and IB clinical trial, with 24 subjects tested. 13 JRC and/or mountain cedar (MC) allergic subjects enrolled in the Phase IB trial and all of them experienced positive-to-negative conversion of skin test for JRC and MC allergens. At the end of this trial, increased JRC specific IgG and decreased IgE levels were observed (ClinicalTrials.gov Identifier: NCT01707069, manuscript in preparation). We demonstrated herein that the CryJ-LAMP Vaccines are excellent in inducing strong antigen-specific Th1 immune responses and in keeping the IgE antibody production low in a JRC allergic model. We also showed that CD4^+^ T cells are the mediators of the immunological effects.

Type I allergy is characterized by secretion of Th2 cytokines such as IL-4 and IL-5, which mediate the production of allergen-specific IgE and promote the local inflammation [25]. Accumulating evidence indicates that activation of Th1 cells could counterbalance the Th2 mediated allergic responses upon DNA vaccination. For instance, in dust mite allergic models, researchers found that DNA vaccination induced high levels of allergen-specific IgG2a and IFN-*γ* production and reduced allergen-specific IgE levels [26–28]. In a JCP allergic model, Toda et al. showed that four doses of CryJ1 DNA vaccine by IM injection induced CryJ1-specific Th1 responses in mice. Meanwhile, gene gun injection failed to achieve the same effects [29]. In agreement with these studies, our results clearly showed that the CryJ-LAMP Vaccines triggered a robust and long-lasting Th1-type immune response in terms of high allergen-specific IgG2a and low IgE production. Even with multiple allergen exposures, vaccinated mice still exhibited a Th1 dominant antibody response. Thus, we propose that the MOA of LAMP Vaccine is through Th1/Th2 skewing.

In several animal models for DNA vaccine studies, it has been found that CD8^+^ T cells play an essential role in regulating the Th1/Th2 responses and/or the IgE production. For example, in a rat model for dust mite induced allergy, Hsu et al. demonstrated that DNA vaccination suppresses the airway hyperresponsiveness by preventing the induction of IgE and the suppression is mediated by CD8^+^ T cells [26]. Gurunathan et al. found that antigen-specific IFN-*γ* producing CD8^+^ T cells are required for the maintenance of Th1 response induced by DNA vaccination in a* Leishmania major* mouse model [30, 31]. Here we showed that transfer of CD4^+^, but not CD8^+^, T cells from vaccinated mice is responsible for the effect of DNA vaccination. Thus, although very likely being activated by vaccination, CD8^+^ T cells by themselves do not directly change the IgG profile and might play a dispensable role in this JRC allergy model. On the other hand, the effects of CryJ-LAMP Vaccines on CD4^+^ T cells are long-lasting as high predominant IgG2a titers and low level of IgE production last over 24 weeks in the recipients of CD4^+^ T cell transfer. Although the detailed mechanisms need to be further elucidated, we propose that specific Th1 effector memory cells are induced upon DNA vaccination. After allergen exposure, rapidly expanded antigen-specific Th1 cells help B cells produce IgG2a antibody and inhibit the Th2 response. As a result, the IgE production remains low.

In a dust mite allergic model, Tan et al. compared DNA vaccines with and without a targeting sequence of LAMP-1 and found that, without LAMP-1, a Th2 response was induced. However, a strong Th1 response was induced when LAMP-1 was included in the dust mite DNA vaccine [27]. Other lysosomal targeting proteins, such as lysosomal integral membrane protein II (LIMP II) and invariant Chain (Ii), have also been utilized to generate DNA vaccines. For example, DNA vaccine encoding the invariant Chain (Ii) and a T cell epitope of CryJ2 protein had been shown to suppress the anti-CryJ2 IgE response in mice [32]. In line with these findings, here we showed the effects of CryJ-LAMP Vaccines in priming robust Th1 responses while suppressing IgE production.

Tregs play a critical role in maintaining immune tolerance. Tregs function through cell contact or secretion of cytokines, including IL-10 [33, 34]. It has been found that IL-10 secreting Tregs are the predominant cells in responding to allergens in the healthy individuals, whereas the Th2 T cells are the major allergen responding cells in allergic patients [35]. Patients with increased numbers of CryJ1-specific Tregs exhibited improved disease symptoms in a clinical trial of sublingual immunotherapy for JCP [36]. In an animal model for birch pollen allergen, Weinberger et al. recently demonstrated that DNA vaccines, which utilized LIMP II to enhance the MHC class II presentation, induced FoxP3^+^ Tregs, but not Th1 cells [37]. In contrast, we found that neither IL-10R blockage nor depletion of CD4^+^CD25^+^ natural Tregs changes the Th1 skewing, suggesting the CryJ-LAMP Vaccines function through a Treg/IL-10 independent mechanism. However, whether inducible Tregs are activated by CryJ-LAMP Vaccines and what role they play in the MOA of LAMP Vaccines are worth further investigation.

Unlike results generated from animal models, human subjects usually produce little or no antibodies by only DNA vaccination [38–41]. It might be true that the DNA vaccines have a low potency of inducing humoral responses in human* per se*. However, recent studies indicate that DNA vaccines are excellent in priming both cellular and humoral immunity, if followed with a protein boost [42, 43]. In this study, protein boosts were administrated to DNA vaccinated animals to mimic an allergen challenge. DNA vaccination elicited detectable levels of allergen-specific IgG antibodies. However, the production of IgG, particularly IgG2a, antibodies was dramatically increased after the allergen exposure. This DNA priming/protein boost like regimen demonstrated its efficacy in inducing high titers of Th1-type antibodies and high levels of IFN-*γ* production. Therefore, we propose that, in future clinical studies, CryJ-LAMP-Vax vaccinated subjects could produce a high level of JRC specific IgG4 antibodies after being exposed naturally to the JRC pollen during the blossom season.

It has been found that the transfection efficiency of the traditional needle injection for DNA plasmid is poor. Therefore, researchers in the DNA vaccine field have pursued better methods to deliver the DNA vaccines. We are particularly interested in the gas-pressured Biojector technique because of its convenience and consistency of administration, as well as its demonstrated efficacy of DNA delivery and transfection [44, 45]. Indeed, we found that needle based administration is capable of inducing high titers antibody production; however, a longer time is needed. On the other hand, Biojector delivery induced Th1-type antibody response rapidly, indicating that DNA vaccines delivered by Biojector have a better dispersion in the injection site, so that more local antigen-presenting cells have access to the DNA plasmids. Furthermore, we delivered the two vaccines together or separately and found no significant difference, suggesting that, in a clinical setting, we may be able to give patients two or more plasmids in one injection. Together these findings provided us with a solid foundation for optimizing the CryJ-LAMP for future clinical application in JCP patients.

## 5. Conclusion 

In this study, our results indicate that the novel LAMP-based DNA vaccines are effective in the mouse model for JRC allergy in induction of Th1-type cellular and humoral responses. CryJ-LAMP Vaccines keep the IgE production in a low level after repeating allergen exposures, indicating this strategy has a potential for clinical application.

## Supplementary Material

Supplementary Figure S1. CryJ-LAMP DNAs delivered by ID injection induce high levels of IgG2a antibody production.
Supplementary Figure S2. CryJ-LAMP DNA vaccines induce antigen specific immune responses.
