# Supplementary material for: CryJ-LAMP DNA Vaccines for Japanese Red Cedar Allergy Induce Robust Th1-Type Immune Responses in Murine Model
Source: J Immunol Res. 2016 Apr 30;2016:4857869. doi: 10.1155/2016/4857869 (PMC4867073; doi:10.1155/2016/4857869)
Supplement: Supplementary file 1 — Supplementary Figure S1. CryJ-LAMP DNAs delivered by ID injection induce high levels of IgG2a antibody production. Supplementary Figure S2. CryJ-LAMP DNA vaccines induce antigen specific immune responses. [file 4857869.f1.zip › Su et al Supplementary Figure 2.pptx]

## Slide 1
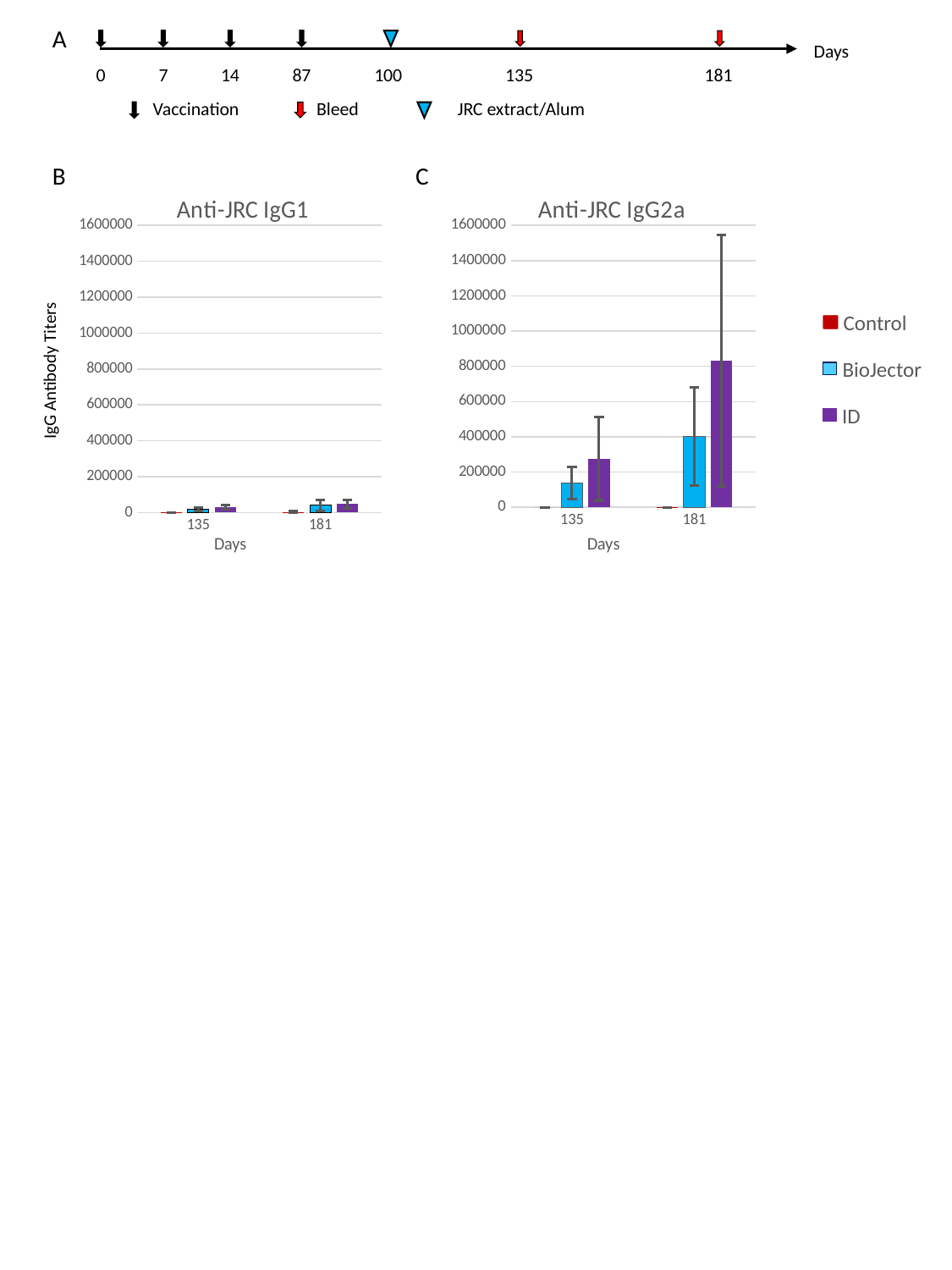

A
100
0
7
14
87
Days
135
181
Vaccination
Bleed
JRC extract/Alum
B
C
### Chart: Anti-JRC IgG1
| Category | Control | BioJector | ID |
|---|---|---|---|
| 135 | 1200.0 | 16800.0 | 29333.333333333332 |
| 181 | 4000.0 | 40000.0 | 47333.333333333336 |
### Chart: Anti-JRC IgG2a
| Category | Control | BioJector | ID |
|---|---|---|---|
| 135 | 0.0 | 136800.0 | 277000.0 |
| 181 | 400.0 | 403200.0 | 831333.3333333334 |Control
BioJector
ID
IgG Antibody Titers
