# Supplementary material for: CryJ-LAMP DNA Vaccines for Japanese Red Cedar Allergy Induce Robust Th1-Type Immune Responses in Murine Model
Source: J Immunol Res. 2016 Apr 30;2016:4857869. doi: 10.1155/2016/4857869 (PMC4867073; doi:10.1155/2016/4857869)
Supplement: Supplementary file 1 — Supplementary Figure S1. CryJ-LAMP DNAs delivered by ID injection induce high levels of IgG2a antibody production. Supplementary Figure S2. CryJ-LAMP DNA vaccines induce antigen specific immune responses. [file 4857869.f1.zip › Su et al Supplementary Figure 3.pptx]

## Slide 1
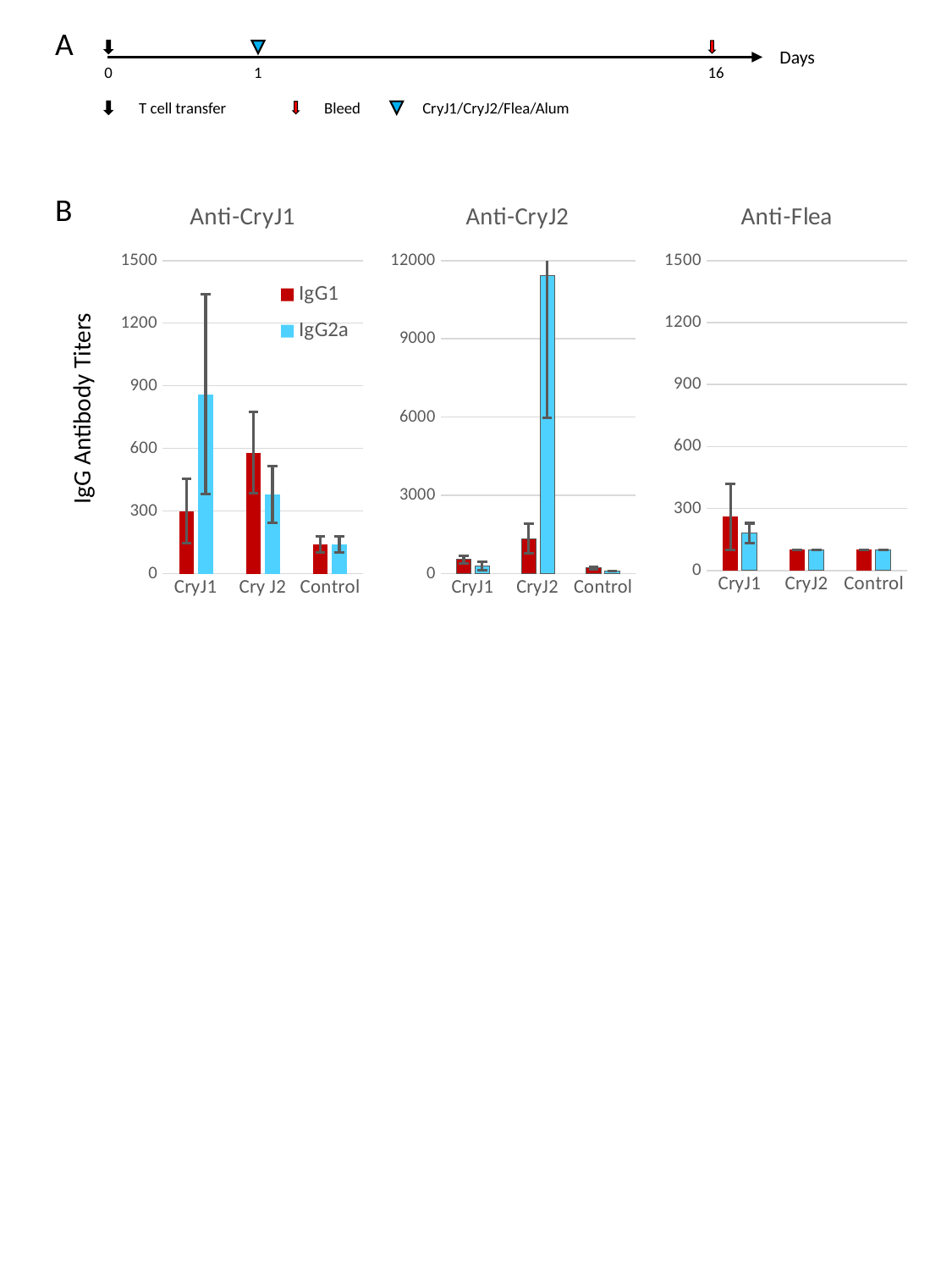

A
Days
0
1
16
T cell transfer
Bleed
CryJ1/CryJ2/Flea/Alum
B
### Chart: Anti-CryJ1
| Category | IgG1 | IgG2a |
|---|---|---|
| CryJ1 | 300.0 | 860.0 |
| Cry J2 | 580.0 | 380.0 |
| Control | 140.0 | 140.0 |
### Chart: Anti-CryJ2
| Category | IgG1 | IgG2a |
|---|---|---|
| CryJ1 | 540.0 | 300.0 |
| CryJ2 | 1340.0 | 11420.0 |
| Control | 220.0 | 100.0 |
### Chart: Anti-Flea
| Category | IgG1 | IgG2a |
|---|---|---|
| CryJ1 | 260.0 | 180.0 |
| CryJ2 | 100.0 | 100.0 |
| Control | 100.0 | 100.0 |IgG Antibody Titers
