# Supplementary figures and images for: CryJ-LAMP DNA Vaccines for Japanese Red Cedar Allergy Induce Robust Th1-Type Immune Responses in Murine Model
Source: J Immunol Res. 2016 Apr 30;2016:4857869. doi: 10.1155/2016/4857869 (PMC4867073; doi:10.1155/2016/4857869)

## Slide 1
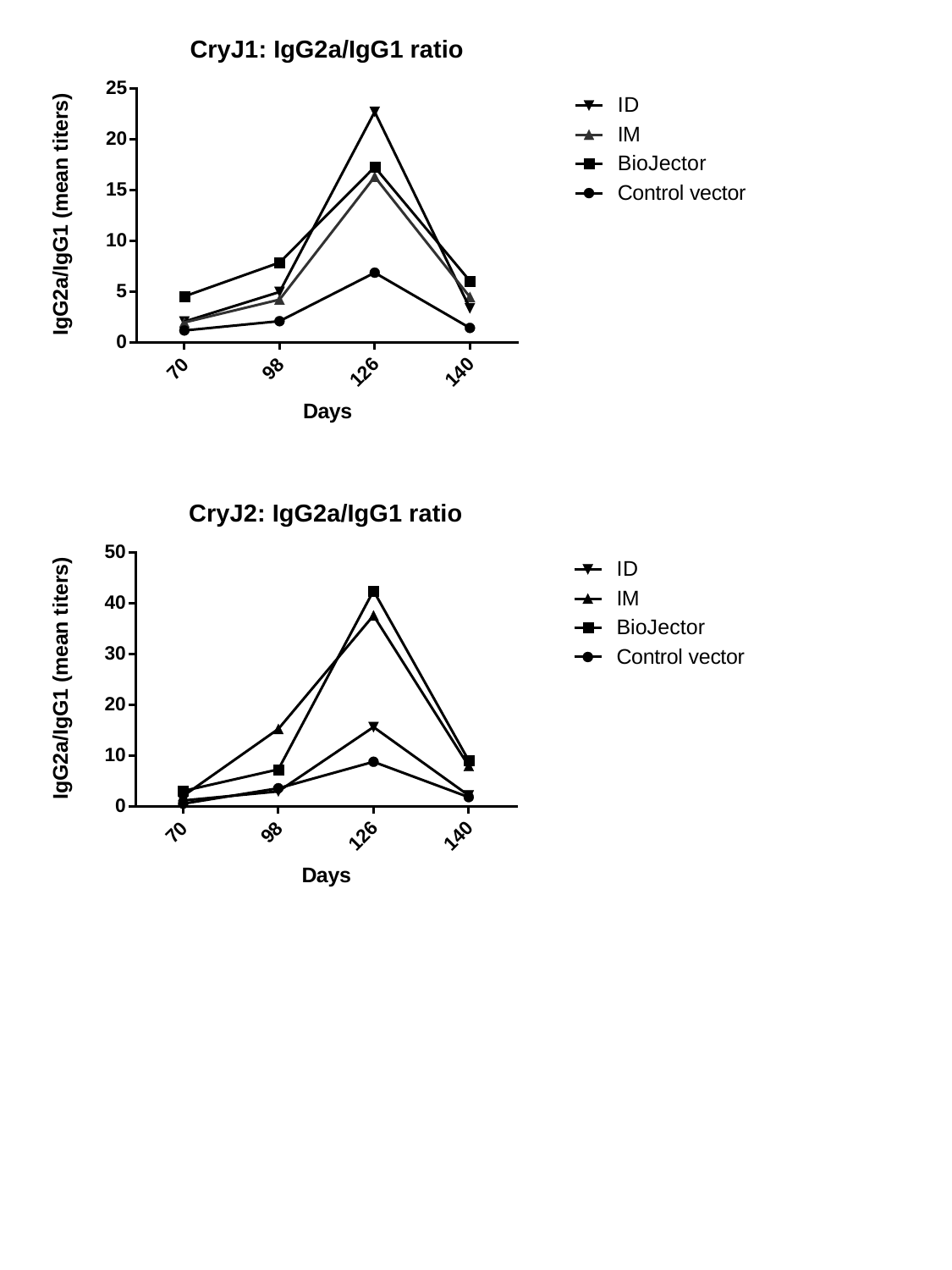

Supplement: Supplementary file 1 — Supplementary Figure S1. CryJ-LAMP DNAs delivered by ID injection induce high levels of IgG2a antibody production. Supplementary Figure S2. CryJ-LAMP DNA vaccines induce antigen specific immune responses. [file 4857869.f1.zip › Su et al Supplementary Figure 1.pptx]

## Slide 1
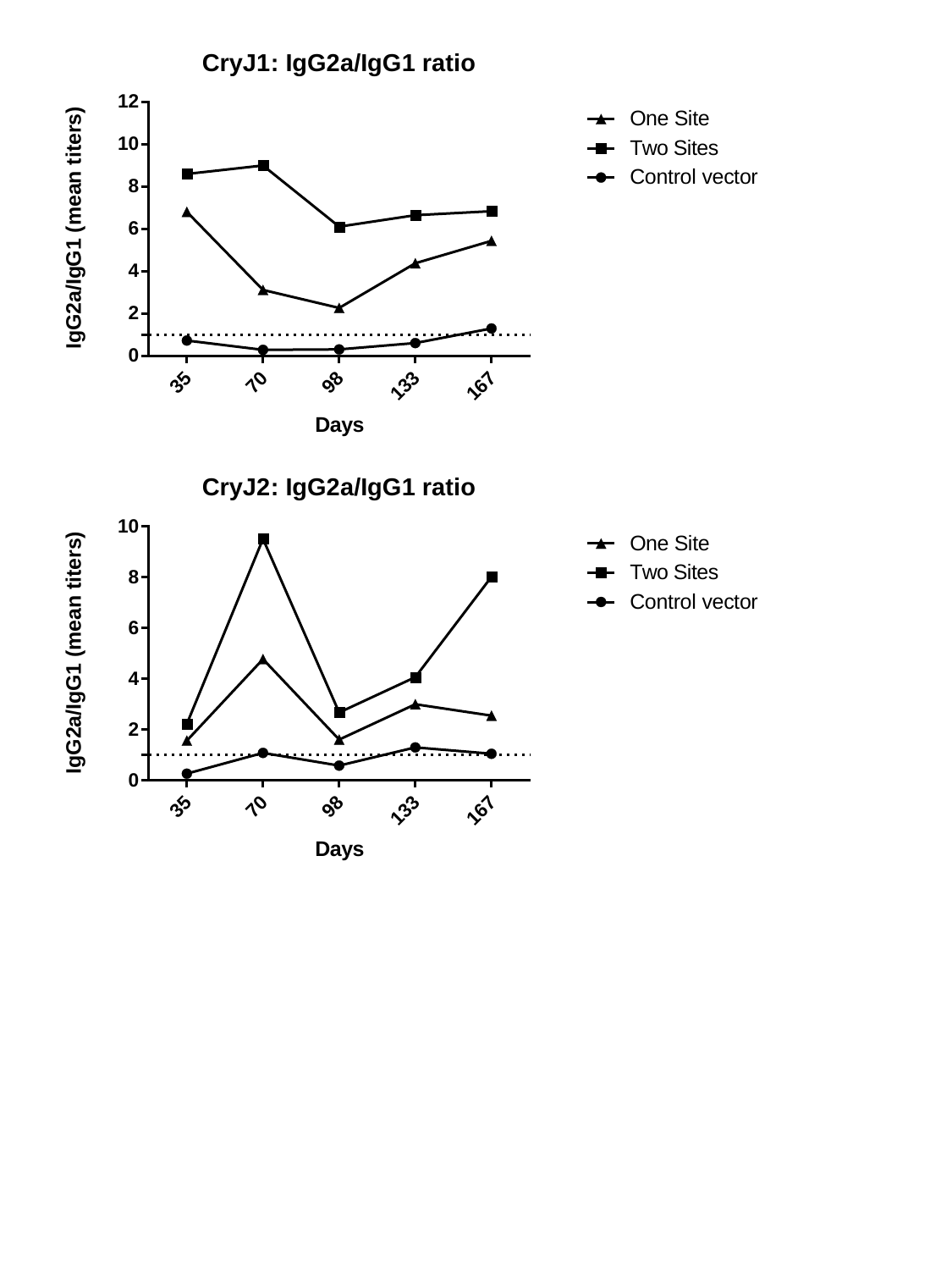

Supplement: Supplementary file 1 — Supplementary Figure S1. CryJ-LAMP DNAs delivered by ID injection induce high levels of IgG2a antibody production. Supplementary Figure S2. CryJ-LAMP DNA vaccines induce antigen specific immune responses. [file 4857869.f1.zip › Su et al Supplementary Figure 4.pptx]

## Slide 1
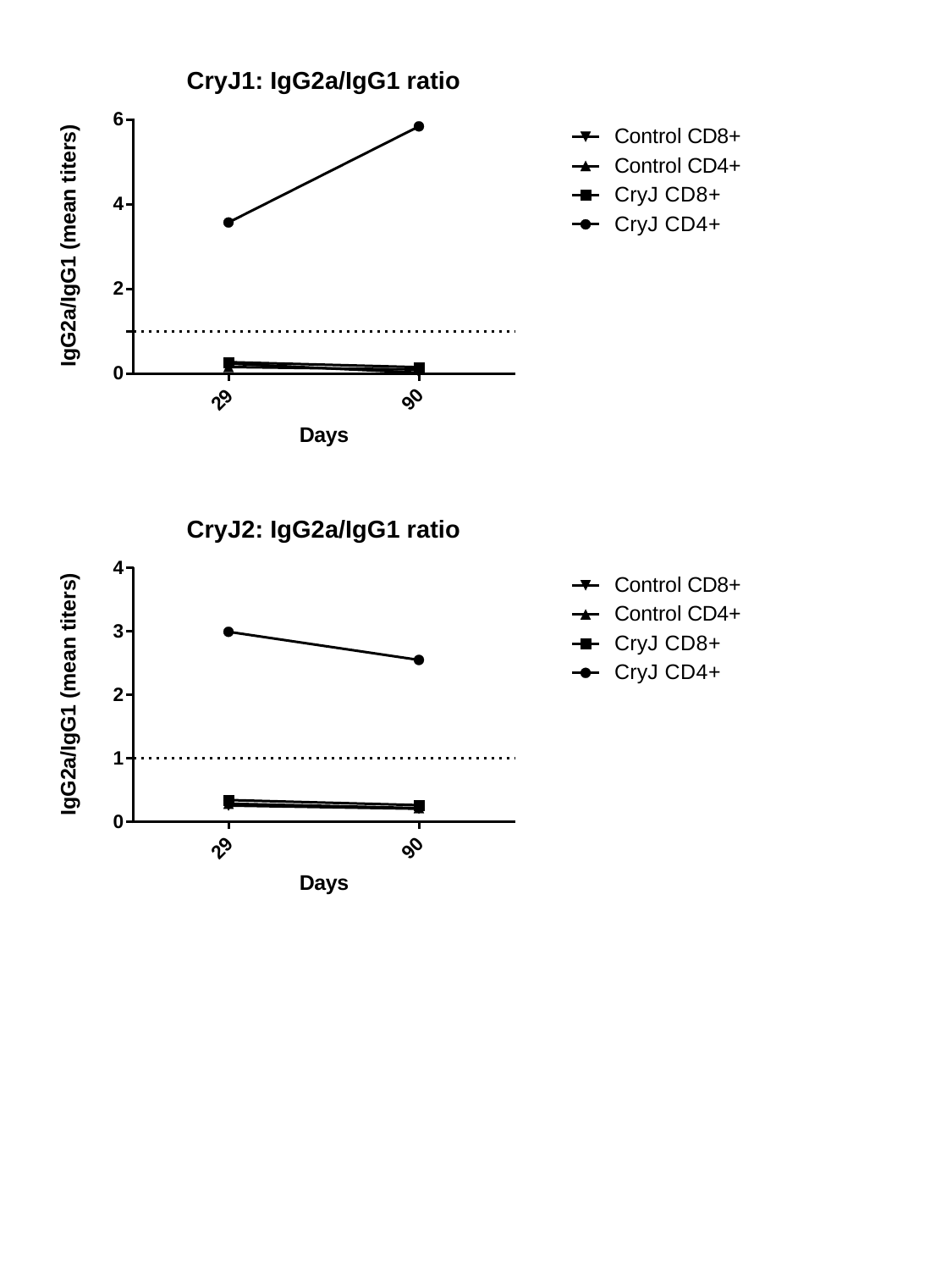

Supplement: Supplementary file 1 — Supplementary Figure S1. CryJ-LAMP DNAs delivered by ID injection induce high levels of IgG2a antibody production. Supplementary Figure S2. CryJ-LAMP DNA vaccines induce antigen specific immune responses. [file 4857869.f1.zip › Su et al Supplementary Figure 5.pptx]
